# Pandemic or Not, Worker Subjective Wellbeing Pivots About the Living Wage Point: A Replication, Extension, and Policy Challenge in Aotearoa New Zealand

**DOI:** 10.3389/fpsyg.2022.828081

**Published:** 2022-05-17

**Authors:** Stuart C. Carr, Jarrod Haar, Darrin Hodgetts, Harvey Jones, James Arrowsmith, Jane Parker, Amanda Young-Hauser, Siautu Alefaio

**Affiliations:** ^1^School of Psychology, Massey University, Auckland, New Zealand; ^2^Department of Management, Auckland University of Technology (AUT), Auckland, New Zealand; ^3^School of Management, Massey University, Auckland, New Zealand

**Keywords:** minimum wage, living wage, pandemic, job attitude, wellbeing, decent work

## Abstract

Recent pre-pandemic research suggests that living wages can be pivotal for enhancing employee attitudes and subjective wellbeing. This article explores whether or not the present COVID-19 pandemic is impacting pivotal links between living wages and employee attitudes and subjective wellbeing, with replication indicating robustness. Twin cohorts each of 1,000 low-waged workers across New Zealand (NZ), one pre- (2018), and one present-pandemic (2020) were sample surveyed on hourly wage, job attitudes, and subjective wellbeing as linked to changes in the world of work associated with the pandemic (e.g., job security, stress, anxiety, depression, and holistic wellbeing). Using locally estimated scatter-point smoothing, job attitudes and subjective wellbeing scores tended to pivot upward at the living wage level in NZ. These findings replicate earlier findings and extend these into considering subjective wellbeing in the context of a crisis for employee livelihoods and lives more generally. Convergence across multiple measures, constructs, and contexts, suggests the positive impacts of living wages are durable. We draw inspiration from systems dynamics to argue that the present government policy of raising legal minimum wages (as NZ has done) may not protect subjective wellbeing until wages cross the living wage Rubicon. Future research should address this challenge.

## Introduction

According to the International Labour Organization (ILO), just prior to COVID-19 the number one challenge in and for the world of work [International Labour Organization (ILO), 2019a] was addressing poor in-work conditions and unliveable wages. According to the ILO in 2019, 3.3 billion people experienced these conditions [International Labour Organization (ILO), 2019b], which was 19 times more than the global unemployment rate (172 million). Two-thirds of the world’s entire workforce was thereby working in conditions that were informal, lacking a proper job description, employment contract, protection in case of injury, regular hours, paid leave provision, social protection, and/or a regular liveable wage. The remaining third were supposed to be protected by a formal, legal Minimum wage floor. However, a survey of 14,000 workers across 14 different countries and economies found that almost two thirds of workers, many in so-called higher-income economies with formal jobs, were “struggling” to make ends meet [International Trade Union Confederation (ITUC), 2018]. Thus the world of work immediately prior to the COVID-19 pandemic could be characterized by in-work precariousness and poverty wages.

Since 2020, the COVID-19 virus has disrupted the whole world of work, and underscored the need for wages worldwide to keep pace much more with people’s everyday needs for decent work conditions that protect their subjective wellbeing, including living wages [International Labour Organization (ILO), 2020, 2021, 2022]. Unlike minimum wages (Smith, 2015), living wages tend to be voluntary rather than statutory and to aim higher than bare subsistence, including affording some disposable income for people to participate with dignity in social life, enjoy occasional treats, and have some financial reserves to buffer them when crises strike. In New Zealand for example, living wages typically include meeting not only material needs like housing and food, but also social needs such as living with dignity and socio-economic inclusion (King and Waldegrave, 2012, 2014). In May 2020, needs like these were recognized in a “wellbeing budget,” which included a commitment to improving wage conditions as part of a broader strategy to promote and protect the wellbeing of the population (New Zealand Treasury, 2020).

Addressing the issue of poverty wages is now very urgent both globally and locally. In 2022, according to the Director-General of the ILO, Guy Ryder, “the global employment and social outlook remains uncertain and fragile” (2022, p. 3), with unemployment being projected to rise (to over 200 m) in 2022, working hours to drop (currently by 2%) and the most vulnerable occupations and smaller organizations within them, hit hardest of all (*ibid*, p. 11). NZ is an example of such a country, yet research on the potential *benefits*, e.g., to subjective and societal wellbeing of raising wages in NZ remains sparse (Carr, 2022). The gap that this paper addresses, and the novelty of its contribution, is to explore the links between wage levels and subjective wellbeing, in the context of a public health crisis.

**Figure 1:**
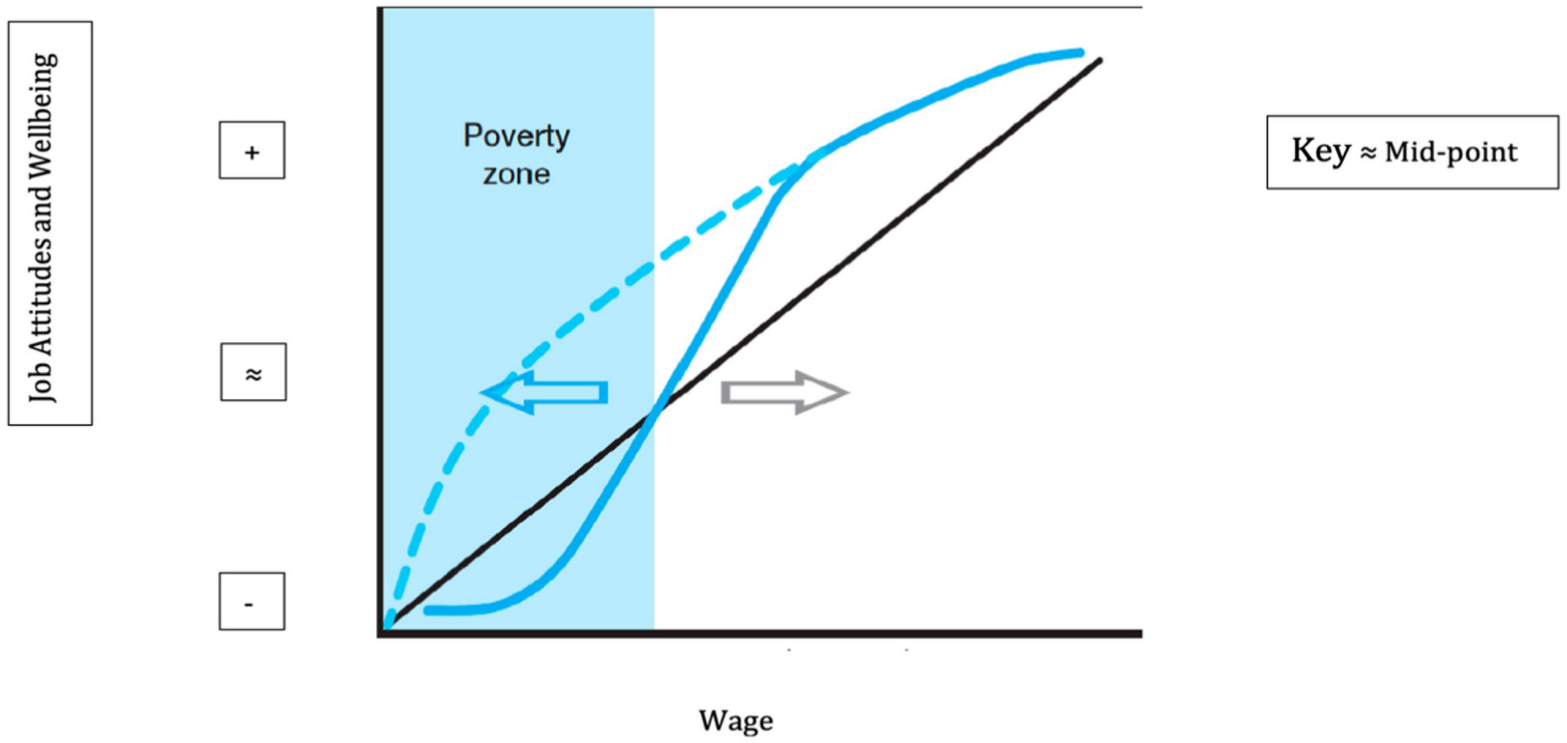
Theoretical links between wage and wellbeing/job attitudes. Adapted with permission from Carr et al. (2016). Wellbeing, in the figure, refers in this paper to subjective wellbeing.

Pre-pandemic, work research on wage and wellbeing has mainly focused on “job attitudes” like job satisfaction and affective commitment, which are organizationally focused, rather than on workers’ wider subjective wellbeing (e.g., Kuvaas, 2006; Judge et al., 2010). Figure 1 presents three theoretical relationships between wage value and job attitudes/subjective wellbeing (Carr et al., 2016). The simplest linkage is linear (black line). Linear implies that there will be a discrete value of wage at which the criteria of subjective wellbeing and job attitudes switch from being negative to positive (≈). Linear linkages have though proved at best disappointingly weak (Young et al., 2014). One reason could be that the link itself may not be linear, for example because wage increments matter more economically, and thus psychologically, at lower than higher wage levels (George and Brief, 1989).

One way in which wage may matter more for lower waged roles is shown in Figure 1 as a blue dotted line. This arc is based on a theory of diminishing marginal returns (Carr et al., 2016). Any wage is a good wage, and especially so at lower levels, where any kind of increment, most of all from zero to one, would make a just noticeable difference. Any resulting curvilinear relationship would thereby be more consistent with reforming minimum, rather than reaching living wages, with minimum cost and thereby lowered risk of job losses during economic and health crises like the COVID-19 pandemic (Law, 2020).

In contrast, working poverty trap theory predicts that workers paid any wage that was less than a viable or living wage, regardless of level, will be stuck at consistently low or negative levels of job attitudes and subjective wellbeing (Carr et al., 2016). This stasis in negative terrain is captured as a flattened section of the curve in Figure 1 at the bottom left of the continuous blue line. Tracking left to right, the solid blue curve begins to rise and eventually crosses the threshold between negative and positive job attitudes/subjective wellbeing (≈) at the top right in Figure 1. Any living wage would thereby need to be set at or near to the monetary value on the *x*-axis where the curve begins to cross ≈ on the *y*-axis. Only from there onwards would it produce diminishing marginal returns.

The last two linkages have not yet—to the best of our knowledge—been fully tested against each other, or been corroborated with systematic evidence. This issue is very important to resolve because of the competing predictions about where to set wages if working poverty is to be avoided and subjective wellbeing enhanced. In Figure 1, diminishing marginal returns (the dotted line) would suggest that there is no working poverty trap. Instead, it suggests that any increase in the minimum wage would make a noticeable difference to job attitudes and worker subjective wellbeing. More importantly still perhaps, the point at which people’s attitudes and subjective wellbeing change valence, from negative to positive, unhappiness to happiness and ill-being to well-being (≈) could be set lower than it would need to be set if there was a working poverty trap (sigmoidal function and solid blue curve).

Of the three theoretical linkages in Figure 1, the available evidence to date, though tentative, is most consistent with the solid blue, sigmoidal curve. Samples of low-waged workers from South Africa and New Zealand, conducted prior to the pandemic, have yielded a consistent pattern (Carr et al., 2018, 2019; Haar et al., 2018). Workers at or near the current Minimum wage, in each respective country, reported struggling to make ends meet, as well as tending to report negative job attitudes (job [dis]satisfaction, [dis]empowerment, and [lack of] occupational pride), and expressing a subjective sense of [un]fairness and [poor] quality of life. Only above the current respective living wage did dissatisfaction, disempowerment, and so on tend to change valence to satisfaction, empowerment, and so forth. Nevertheless, the samples in this study have been relatively small and localized, with measures that were also non-standardized. Such concerns led Carr et al. (2018) to call for more systematic study of the three hypothetical links in Figure 1.

A subsequent study, conducted in NZ, included a larger, nationwide sample (*N* > 1,000) and standardized measures (Carr et al., 2019). This study also replicated findings from Carr et al. (2018). It focused on job attitudes (job satisfaction, work engagement, meaningful empowerment, affective commitment, organizational citizenship behaviors, and work life balance). Among these, only job satisfaction and work life balance have links to subjective wellbeing, specifically happiness at work (Fisher, 2009); with a potential for spill-over into respondents’ lives more generally (Haar et al., 2014). Subsequent qualitative analysis of individual ‘outliers’ found signs of mental health issues arising from precarious wage conditions, including stress, anxiety, and depression (Carr et al., 2019). These findings in turn led Carr et al. (2019) to call for further exploration of potential linkages between living wages and mental health.

Public health research on minimum wages has found a range of likely state-level benefits from state-level increases on population wellbeing, including physical (Lenhart, 2017) and mental health (Leigh et al., 2019). Examples would include reductions from diseases of the circulatory system that lead to premature deaths from conditions such as strokes (Tsao et al., 2016) and suicide (Kaufman et al., 2020). However, societal-level studies cannot tell us directly if state-level improvements are only among minimum wage workers or also for people paid more from wage inflation, including among those paid a living wage. Moreover, everyday forms of mental wellbeing at and through work (e.g., job stress, experiences of job insecurity, anxiety, and depression) remain largely unconnected within the relatively new field of wage and subjective wellbeing (Leigh et al., 2019). Triggers for stress-induced strains on subjective wellbeing have included concerns about wages, especially for those below the median income (Chuluun et al., 2016). Additionally, employees who perceive their organization as unfair and feel job insecure may also be at higher risk of emotional exhaustion and work stress (Kausto et al., 2005).

Subjective wellbeing concerns like these, which are due to precarious work and wage conditions, have almost certainly been amplified by the COVID-19 pandemic. In NZ for example, a nation-wide survey of over 3,000 people has found that almost 40 percent of households experienced a significant drop in work-related income (Galicki, 2020). One-in-four households were caught in arrears on at least one payment (including consumer loans, utility bills, and housing costs). One in 10 had missed a rent or mortgage payment. Forty-one percent either agreed or strongly agreed that thinking about their financial situation made them anxious. Anxiety like this, about unmet financial needs, can contribute to a range of related mental health issues, including anxiety and depression (Shaw and Gupta, 2001; Stride et al., 2007). The question then is could a living wage^1^ provide any social protection for people’s mental health, by helping to meet people’s financial needs in NZ during a pandemic?

Pandemics are inherently and intrinsically threatening to both physical and mental wellbeing. This threat renders an assessment of the links between wage and subjective wellbeing, which Carr et al. (2019) were already calling for prior to COVID-19, even more timely and relevant. Pandemic conditions have not only threatened jobs and worker wellbeing. They have also changed the conditions of work itself. In NZ for instance,^2^ overall employment effects of COVID-19 were moderated by substantial public policies directed at subsidizing wages to protect jobs and assisting selected industries and small businesses. There were programs directed at health care, vulnerable groups, and easing risks of mortgage defaults and the eviction of renters. At the same time, there were also substantial declines in hours worked, which reduced annual wages and incomes for many households. These COVID-19 policies and labor market changes conceivably had direct effects on the wellbeing of low-income workers as well as an indirect effect on their observed job attitudes and subjective wellbeing, causing potentially untold compositional changes in the unobserved characteristics of the low-income workers who remained employed and the jobs that they held. In other words, COVID-19 has been a great disruptor.

Disruptors are not only negative or destructive, however. They also bring unique learning opportunities. In research, one of these is the opportunity to see if any given function will replicate even under radically changed circumstances. Any finding that a function in Figure 1 remained similar over radically different circumstances will “strongly attest to its durability across time” (Gergen, 1973, p. 315), by signaling robustness in that function (Schupbach, 1998). Thus a replication of the cusped curve in Figure 1, under disrupted pandemic conditions, *would give confidence that a living wage is pivotal for subjective wellbeing*.

Policy wise, this pandemic has also re-ignited heated and often fractious debate in NZ regarding the need for a living wage (Waldegrave, 2020). This debate is not unique to NZ (e.g., Wood, 1997; Kantor, 2016). On the one hand, advocates of living wages present any wage increase as the right thing to do for social inclusion and shared prosperity (Krieble, 2020). On the other hand, advocates of wage restraint, including some influential employer lobby groups, insist that raising wages to living wage levels, especially during a pandemic and associated economic crisis, will only lead to job losses, more job insecurity, and even less social inclusion (Law, 2020). To date, however, this debate has largely overlooked both job attitudes and worker subjective wellbeing, which are important considerations that have been linked to work productivity, at both individual (*per capita*) and unit (aggregated organizational and sub-organizational) levels (respectively, Harter et al., 2002 and Harrison et al., 2006). Evidence on how wage relates to worker job attitudes and subjective wellbeing might therefore cast light on a heated debate about the social *and* economic wisdom of wage increases, precisely at a time of crisis for sustaining livelihoods (United Nations, 2021).

Briefly, the overall objective in this study was to explore whether the sigmoidal relationship or alternatively either of the other two competing relationships in Figure 1 would replicate in NZ. Replicability was assessed in two main ways: (a) across two cohorts, one sampled before and one during the current pandemic; and (b) across two sets of variables, one focused on different job attitudes and the other more on humanitarian features of people’s everyday subjective wellbeing.

The overarching aim of this study was to assess if the concept of a living wage has practical and policy implications. Taking an evidence-based approach, we wanted to know whether there was any consistently identifiable, actual material wage value whereabouts workers in NZ would tend to report feeling better, not only about their work attitudes, but also in their wider subjective wellbeing.

## Materials and Methods

### Participants

Participants were drawn from two national cohorts of lower income workers, one during March/early April 2018 (*n*_1_ = 1,011) and another in September/October 2020 (*n*_2_ = 1,027). All respondents had to be in paid employment with an annual personal income before tax of under NZ$60,000. A professional survey organization, Qualtrics, was engaged to draw two samples from across NZ, one in 2018 and a second in 2020 (Haar et al., 2018). The Qualtrics system has an estimated time for surveys, and removes respondents who complete the survey too quickly or too slowly. It also assures that one respondent only can complete the survey. This approach to data collection has grown and provided useful samples for researchers (Ferguson et al., 2014; Kaplan et al., 2016; Vitell et al., 2016). We utilized this approach specifically because Qualtrics can target income-level within their respondent recruitment. It pays respondents for their time, but the nature of this arrangement is proprietary.

In the 2018 cohort, by income level, the modal reported annual income, expressed in brackets of NZ$20,000 s, was NZ$40–60,000 band (39% of sample), followed by NZ$20–40,000 (36%), and then up to NZ$20,000 (25%). Most workers were paid hourly (71%) rather than being salaried (29%), 86% with one job, and working full-time (51%). Our lower-waged sample was skewed toward female workers (69%), with the modal age category being 36–45 years (19%). Ethnically, the majority reported as “NZ European” (62%) with the next largest category identifying as Māori (11%). By sector, the majority worked in the private sector (68%) rather than in either public service (17%) or in civil society organizations like Non-Government Organizations (NGOs) and charities (15%). These proportions have already been found to be reasonably representative of the lower-end of the wage spectrum and economy across NZ at the time (Carr et al., 2019).

In the 2020 cohort, the modal income level was NZ$40–60,000 (50%), followed by NZ$20–40,000 (34%) and up to NZ$20,000 (17%). A majority of workers (66%), as in 2018, were paid hourly (66%), 86% with one job, and working full-time (53%). As in the 2018 cohort, there was a skew toward female workers in this low-waged sample (65%), with the same modal age category (of 36–45 years, 22%). The two top ethnicities were NZ European and Māori (respectively, 65 and 12%). Private sector work was once more predominant (67%) over public service (20%) and civil society (13%).

### Measures

In addition to a range of standard demographic items (Haar et al., 2018; Carr et al., 2019), we focused in this paper on three particular sets of variables, reflecting (i) wage level, (ii) job attitudes, and (iii) subjective wellbeing. These were examined separately over the two cohorts in 2018 and 2020.

#### Wage

Hourly pay and annual income, number of paying jobs, and full or part-time employment (*cf.*, Carr et al., 2019). In 2020 only, we asked whether during the preceding maximum level 4, full lockdown people had been able to work from home, had a pay cut or bonus, experienced a temporary layoff, and cuts to pay (including hours) and whether these were back to normal. We also asked if cost-of-living (during lockdown) went up or down.

#### Job Attitudes

From Table 1, we measured Job satisfaction using three-item (Judge et al., 2005; *α* = 0.91, 0.91); work engagement on nine-item omnibus measure of Schaufeli et al. (2001)(*α* = 0.92, 0.92); career satisfaction with three items of Greenhaus et al. (1990) (*α* = 0.85, 0.87); meaningful work with three items from Spreitzer (1995) (*α* = 0.93, 0.91); affective commitment with three items from Meyer et al. (1993); (*α* = 0.78, 0.76); organizational citizenship behaviors (OCBs) using four items from Lee and Allen (2002) (*α* = 0.84, 0.82); and work-life balance with three items from Haar (2013) (*α* = 0.88, 0.86). In 2018, these measures were subjected to one combined confirmatory factor analysis (CFA; Haar et al., 2018). We used (1) the comparative fit index (CFI ≥ 0.95), (2) the root mean square error of approximation (RMSEA ≤ 0.08), and (3) the standardized root mean residual (SRMR ≤ 0.10). The combined CFA confirmed that our measures were each internally coherent, distinctive from each other and relatively free of common method bias (for details, Haar et al., 2018).

**Table 1:** Key psychological measures.

Construct	Source	Exemplar item
Job Attitudes		
Job Satisfaction	Judge et al., 2005	“I find real enjoyment in my work”
Work Engagement	Schaufeli et al., 2001	“I am proud of the work that I do”
Career Satisfaction	Greenhaus et al., 1990	“I am satisfied with the success I Have achieved in my career/work”
Meaningful Work	Spreitzer, 1995	“My job activities are meaningful to me”
Affective Commitment	Meyer et al., 1993	“I would be very happy to spend the rest of my career with this organization”
Organizational Citizenship Behavior (OCB)	Lee and Allen, 2002	“I assist others with their duties”
Work-Life Balance	Haar, 2013	“I manage to balance the demands and personal/family life equally well”
Subjective Wellbeing		
Job Security	Armstrong-Stassen, 2001	“I am worried about being laid off”
Job Stress	Stanton et al., 2001	“Overall, how would you rate your stress from 0 to 10?”^*^
Anxiety	Axtell et al., 2002	“Calm—Never…. Always[five-points]”^*^
Depression	Axtell et al., 2002	“Optimistic—Never-Always…”^*^
Holistic Wellbeing	Tomlyn and Cummins, 2011	“How satisfied are you with Your health?”

* Reverse-scored.

#### Subjective Wellbeing

Job security was measured using a three-item measure from Armstrong-Stassen (2001) (*α* = 0.88, 0.92); job stress using a single item in Stanton et al. (2001); [“Overall, how would you rate your stress from 0 (no stress) through 5 (neutral) to 10 (extreme stress)?”]; anxiety and depression with three items each from Axtell et al. (2002) (respectively, *α* = 0.92, 0.92; *α* = 0.92, 0.91). Holistic subjective wellbeing was assessed on a wide-ranging physical-mental-spiritual 10-item measure in Tomlyn and Cummins (2011) (*α* = 0.91; 0.92).

### Measurement Models

Using Analysis Of Moment Structures (AMOS) version 26, we conducted a pair of CFAs for each cohort on the job attitudes and subjective wellbeing constructs. We followed Williams et al. (2009) regarding assessing model fit: (1) the comparative fit index (CFI ≥ 0.95), (2) the root-mean-square error of approximation (RMSEA ≤ 0.08), and (3) the standardized root mean residual (SRMR ≤ 0.10). Overall, from Table 2, the hypothesized measurement model was the best fit for the data meeting all minimum thresholds job attitudes We ran alternative CFAs (combining various constructs) and these all resulted in poorer fit models (all *p* < 0.001; Hair et al., 2010).

**Table 2:** Results of confirmatory factor analysis (CFA).

	*χ^2^*	*df*	CFI	RMSEA	SRMR
*Job Attitudes*					
2018	1301.9^*^	328	0.95	0.05	0.05
2020	1257.9^*^	328	0.96	0.05	0.05
*Subjective Wellbeing*					
2018	896.5^*^	161	0.95	0.07	0.04
2020	1012.1^*^	161	0.95	0.07	0.04

* *p* < 0.001.

### Procedure

This project was funded by the Royal Society of NZ (RSNZ) Marsden Fund (17-MAU/137). Ethical approval was obtained from Massey University Human Ethics Committee (MUHEC) (2018). All participants were assured of confidentiality and remained anonymous to the researchers. As noted under “Participants,” the survey was designed by the authors and distributed *via* a private research company, Qualtrics. We utilized this approach because Qualtrics can target income level in respondent recruitment and because their respondents are already familiar with surveys. Respondent familiarity may have introduced a familiarity bias (with survey forms), but this is offset by the possibility of using multiple items (more familiar to such panels), increasing reliability and validity (Carr et al., 2019). During piloting, lack of familiarity was identified as a barrier to participation by lower-income groups (Haar et al., 2018).

A first survey took place before the pandemic began, in the first quarter of 2018. A second survey took place after the maximum level, full “level 4” lockdown from March 31, 2020 to April 27, 2020 had ended and life (including work life) had pretty much returned to normal. These twin survey cohorts hence straddled pre-pandemic and pandemic.

## Results

Following Carr et al. (2018), we first used curve estimation to explore the best-fitting function (line, logarithmic curve, or sigmoid/cubic, as in Figure 1) to the data for each job attitude and subjective wellbeing measure, as a function of hourly wage. In both cohorts, hourly wage was clearly the modal means of payment, not annual salary (cohort 1 *n* = 722; cohort 2 *n* = 680). Accordingly, we again focused mainly on this form of payment. In 2020, we used current (COVID-affected) rather than normal hourly rate (which differed for *n* = 25 respondents). Within cohort 1 and 2, there were potentially distorting wage outliers that ranged from NZ $0/h at one extreme to $2,050/h at the other. In order to maintain consistency, we selected cases who were paid anywhere from legal adult minimum hourly wage at the time hour up to and including $40/h (≈$60,000/annum for 30 h/week).

Across job attitudes, there was a pattern that replicated across the changed circumstances in cohorts 1 and 2 (Table 3). First, job attitudes covaried significantly (*p* < 0.01) with hourly wage. Second, the link was non-linear (the only partial exception being OCB). Third, the form of curve was predominantly sigmoidal not logarithmic, resembling the solid curve in Figure 1 (Carr et al., 2016). There were three instances where the precise nature of the curve was inconclusive, at this stage.

**Table 3:** Curve estimations for Job Attitudes in cohorts 1 and 2.

Attitude	Cohort	Best fit	*F* value	*df*	Percent variance
Job Satisfaction	1	Cubic	10.16^****^	3,423	6.7
	2	Cubic	10.05^****^	2,445	4.3
Work Engagement	1	Cubic	7.42^****^	3,423	5.0
	2	Cubic	5.86^***^	2,445	2.6
Career satisfaction	1	Cubic	13.78^****^	3,423	8.9
	2	Cubic	14.14^****^	2,445	6.0
Meaningful work	1	Cubic	12.95^****^	3,423	8.4
	2	Cubic	9.07^****^	2,445	3.9
Affective commitment	1	Cubic	7.72^****^	3,423	5.2
	2	Cubic	7.88^****^	2,445	3.4
OCB	1	Cubic/Logarithmic/Linear^****^		Tied	4.6
	2	Cubic/Linear^****^		Tied	4.6
Work Life Balance	1	Cubic	3.86^**^	3,423	2.7
	2	Cubic/Logarithmic^****^		Tied	5.0

** *p* < 0.01;

*** *p* < 0.005;

**** *p* < 0.001.

Variances explained in Table 3. Though well within normal limits in psychological research, these were not high. This could be partly due to the cusped nature of sigmoidal functions, to which curve estimation may not be sensitive. To explore that possibility further, we applied Locally Estimated Scatterplot Smoothing (LOESS). LOESS is more sensitive than curve estimation to points of inflexion (Carr et al., 2019).

**Figure 2:**
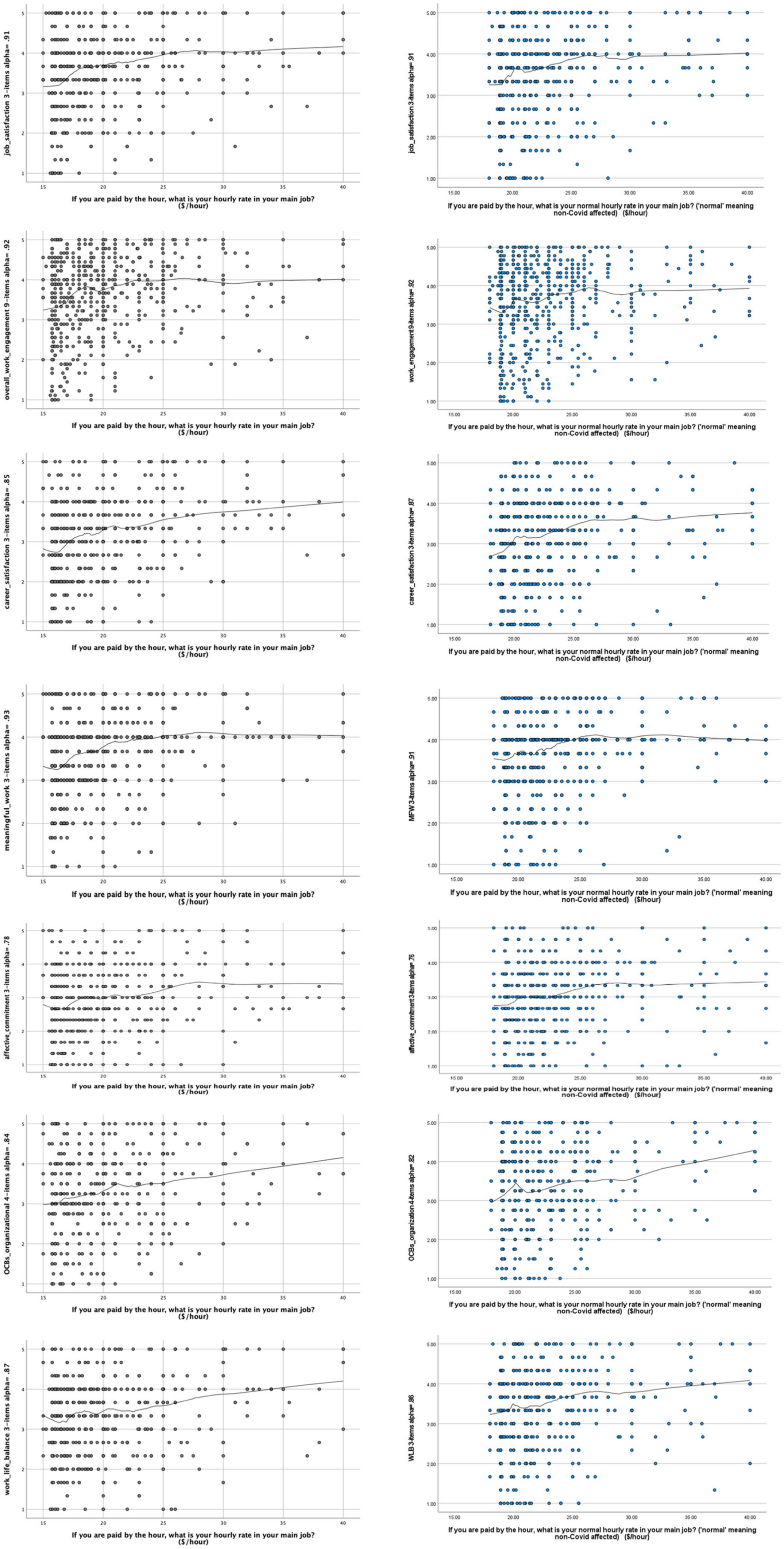
Locally Estimated Scatterplot Smoothing (LOESS) curves for Job Attitudes (*N* = 593 + 619 = 1,212, tension parameter = 0.35). We reset the range of sampled wage values to include legal Minimum wage and above (*n* = 21 excluded).

From Figure 2, there was a consistent pattern, with the lowest-waged workers tending to score relatively low on job attitudes. This is seen in a clear wage-subjective wellbeing tail, in which there was little or no increase in job attitude score for increments in wage. In other words, there was a consistent working poverty trap. Comparing the two time periods left and right in Figure 2, the curve consistently pivoted from flat to zero to positive gradient, flat to upward, and peaked first about $NZ20 per hour (the NZ Living wage in 2018 and 2020, respectively, was $20.55 and $22.10 per hour). Thereafter, there tended to be a slight dip, followed by a curve indicative of diminishing marginal returns (just noticeable differences). Thus, for job attitudes, the data summarized in Table 3 and Figure 2 point convergently to the solid blue curve in Figure 1.

**Figure 3:**
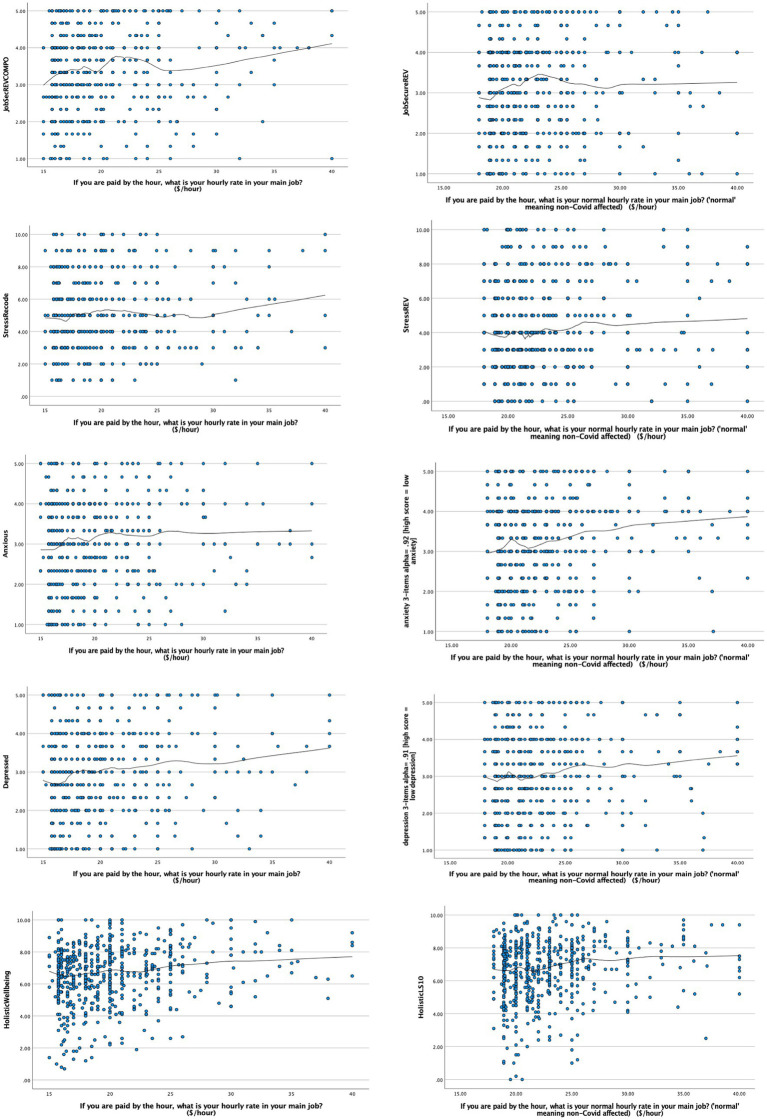
LOESS curves for Subjective Wellbeing (*N* = 1,212, tension parameter = 0.035–40).

Curve estimations for subjective wellbeing variables were increasingly distal from the workplace itself (Table 4). Accordingly, the percentages of variance explained were less than in Table 3. Non-linear relationships still predominated over linear linkages, although the precise nature of the curvature (logarithmic or cubic) was more often inconclusive. Again therefore, we utilized LOESS regression analysis, which were more capable of detecting any potential cusp-like inflexions in the curve, which had not been detected using curve estimation. LOESS curves charting the links between hourly wage on the one hand and subjective wellbeing indicators on the other, in each respective cohort (i.e., during 2018 and 2020) are presented in Figure 3.

**Table 4:** Curve estimations for Subjective Wellbeing in cohorts 1 and 2.

Subjective Wellbeing facet	Cohort	Best fit	*F* value	*df*	Percent variance
Job (in)Security	1	Cubic	4.04^**^	3,574	2.1
	2	Cubic	3.77^*^	2,630	1.2
Job Stress	1	Cubic	2.36 (*p* = 0.07)	3,574	1.2
	2	Cubic/Logarithmic		Tied	1.1
Anxiety	1	Cubic	3.46^*^	3,574	1.8
	2	Linear/Logarithmic/Cubic^****^		Tied	2.4
Depression	1	Cubic	5.94^****^	3,574	3.0
	2	Linear/Logarithmic/Cubic^*^		Tied	1.4
Holistic wellbeing	1	Linear/Logarithmic/Cubic^****^		Tied	3.6
	2	Cubic	10.68^****^	2,630	3.3

*p < 0.05; **p < 0.01; ****p < 0.001.

From Figure 3, there was both pattern and a possible slight deviation from pattern. First, the relationships were cusped, with discernible mini peaks at around NZ$20 per hour. A visible exception to these consistent patterns was the curvature for job (in)security, which peaked nearer $25/h. The threshold for job security may be understandably higher during an economically uncertain pandemic.

*Post hoc*, we conducted a check on this level of uncertainty in 2020 (Materials and Methods). Almost half the full sample (47%) could not work from home during the full lockdown period, which rose to fully 76% for workers paid by the hour. One third of workers had experienced some form of pay cut during the lockdown itself. One-in-five had been temporarily laid off, among whom 40% had not received any wage subsidy. Only 13%–14% of cohort/hourly-paid workers had received a wage bonus. By the time of the survey that was taken in 2020, the overall pay rate was back-to-normal for 87/88% of the cohort/hourly-paid. Where it was not, waged hours were implicated. Among employees paid by-the-hour, approximately one-in-three reported a cut in their waged hours during lockdown, with the number of paid hours still not having returned to normal for 20 percent of them. During the lockdown, cost-of-living rose for 30% of the cohort, and fell for 26%, but remained the same for 40% of the overall sample.

## Discussion

In terms of theory, our findings were most consistent with the concept of a workplace poverty trap, meaning the need for wages to exceed a certain threshold value before job attitudes AND subjective wellbeing are properly safeguarded (Krieble, 2020). Looking back at Figure 1, the findings in this extended study are in general more consistent with the blue sigmoidal curve than with the concept of diminishing marginal returns (or just noticeable differences). According to this rival perspective, any job is a good job, and any wage is a good wage (Law, 2020). In our study, across both waves, and both different circumstances, just noticeable differences (JNDs) only became apparent not before, but rather *after* the living wage threshold was crossed (Carr et al., 2016). The consistency of this pattern, in which JNDs were more visible only well above the living wage and not below it, is indicative of robustness (Gergen, 1973; Schupbach, 1998). Replicability was sustained through a pandemic, and across from job attitudes into subjective wellbeing. Indeed, comparing Figures 2, 3, the poverty trap zone was visibly more clearly “under” the waterline (≈) in the case of subjective wellbeing than it was for job attitudes. This indicates that reaching the living wage was especially salient for subjective wellbeing.

The amount of variation that we observed about the LOESS curves (Figures 2, 3) was consistent with other studies in NZ (Carr et al., 2018, 2019). It likely reflects the everyday diversity of people’s work and life circumstances (Carr et al., 2021), which includes work (and wider living) conditions other than hourly wage, such as average number of hours worked per week (Carr et al., 2021). In this study, we did not compute job attitudes and subjective wellbeing as a function of hours worked. We used an arguably cruder measure, mainly rate of pay per hour. This was nonetheless the pay modality specified in both the minimum and living wage in NZ. It was also the modal form of pay for our sample of relatively low-waged workers. Although a majority of respondents were employed on a full-time basis, almost half (Method) worked on part-time contracts, with variable hours. Variations in the number of hours worked per week may thereby account for at least some of the spread of data points about the curves marking central tendencies in Figures 2, 3 (and across both hourly-paid and salaried workers). Future research may nonetheless explore hours worked as a moderator of links from pay (hourly and salaried) to job attitudes/subjective wellbeing.

Our research took place in a period when the government of NZ was focused on raising the legal minimum wage to more closely approximate the country’s higher, but also voluntary living wage. The two surveys of cohorts took place approximately 2.5 years apart, from April 2018 to October 2020. During this time, the mandatory legal minimum wage rose from NZ$16.50 to NZ$18.90 per hour (an increase of 14.5%, reflected in the rightward deflection of the lowest wage point in Figures 2, 3 from 2018 to 2020). The living wage rose from $20.55 to $22.20 per hour (an increase of 8%). Overall, between the first and second survey cohorts, the minimum wage made a net gain on the living wage in the order of more than 5%. This gain was exclusive of the rate of price inflation from 2018 to 2020, although the rate was relatively low, at just 1.6% *per annum* for both these years (Statista, 2021).

Despite this gain by the minimum on living wage, Figures 2, 3 show that the NZ$ wage-value at which people tended to report subjective wellbeing *above* the waterline (≈) remained visibly *above* the actual minimum wage value, across both cohorts. Whether subsequent planned minimum wage increases (in 2021, after this study was over) will be sufficient to significantly reduce the poverty trap tail in Figure 1 remains unknown until future tests can be conducted (forthcoming).

In the meantime, a focused wage reform alone may not be enough to eradicate (working) poverty. A wider approach could be required, in which a whole suite of policies, from wages to housing, are reset as part of a wider assemblage (Hopner et al., 2021). COVID-19 has not destroyed infrastructure and installed production capacity, in fact it is disorganizing production chains and changing many aspects of the labor market, as well as aggravating social and economic problems, especially for low-income workers. Policies aimed at social protection have been adopted in several low- and middle-income countries, such as conditional cash transfers, housing programs for the low-income population, free health care, etc., and could mitigate the problem of low-income workers.^3^ These initiatives deserve more applied and evaluative research attention, focused on how they integrate vs. possibly undercut each other to affect people’s everyday work-related subjective wellbeing (Hasdell, 2020).

With respect to wage reforms and addressing in-work poverty in NZ for example, the success of any particular reform may depend not only just on wage reform policy itself, but also on how well the government combats *other* social issues, like housing unaffordability, as part of a wider, more concerted push. For example, if rental prices simply rise ahead of any centrally-implemented rises in the legal minimum wage, then any increases in take-home wages will simply be eroded by the increased cost of housing rental. COVID-19 has put additional strain on people and livelihoods, as well as government coffers. Thus a more systems-wide approach to tackling in-work poverty, going even wider than the current wellbeing budget (New Zealand Treasury, 2020) may still be required—and require to be evaluated in future research.

With respect to future research, our study focused on subjective wellbeing, even though the concept itself is broader, extending to physical health and a range of public health statistics other than those sampled in this study alone (Leigh et al., 2019). We have also conceptualized job attitudes as predominantly related to work performance, when in fact they overlap with subjective wellbeing, for example through the concept of happiness (Fisher, 2009). We chose the indicators we did because they are linked to workplace performance (Carr, 2022). Yet as Fisher (2009) and Haar et al. (2018) have pointed out, happiness in the work place, for instance job and occupational satisfaction often links to life satisfaction, i.e., happiness in wider society. In that sense, future research should sample different facets of wellbeing, and explore the possibility that occupational subjective wellbeing is a *mediator* between wage and wellbeing.^4^

In conclusion, our data were largely subjective, rather than econometric. The study aimed to explore job attitudes and aspects of subjective wellbeing, both inherently experiential and relevant, during a turbulent and disrupted time in NZ (work) history. From that humanitarian work psychology perspective (Carr et al., 2012), the replicability of previous findings, during a pandemic, points toward a certain robustness in the idea of a living wage being pivotal for eradicating poverty. Beyond that point, our findings have also posed a serious challenge to governments like ours in NZ. The challenge is, to not only keep raising minimum wages to meet living wage levels, but also perhaps to make more of a dynamic systems approach—that simultaneously regulates a suite of policy options in the world of work, housing, and other costs of living. This change should be done in partnership with key stakeholders from labor and management (Arrowsmith et al., 2020). It should somehow manage to include other material cost-of-living factors, like housing rental prices, from the wider NZ societal system.

### Practical Implications

1. The government of NZ should keep raising the Minimum wage closer to, and preferably alongside the Living wage.
2. Raises to the Minimum wage will not work unless they are coupled to policy changes to freeze or restrain rents, and to link Minimum wage increases, dynamically, to rising inflation.
3. Evaluations of increases to the Minimum wage should include the health cost savings from boosted wellbeing, subjective, and objective, at work and in society.

## Data Availability Statement

The original contributions presented in the study are included in the article/supplementary material, further inquiries can be directed to the corresponding author.

## Ethics Statement

The studies involving human participants were reviewed and approved by the Massey University’s Northern Human Ethics Committee [Massey University Human Ethics Committee (MUHEC), 2018]. The patients/participants provided their written informed consent to participate in this study.

## Author Contributions

SC, JH, DH, HJ, JA, JP, AY-H, and SA conducted the study, contributed to the research and the writing of the article, led by SC, JH, and DH, and the article was revised and edited by the other research team members. All authors contributed to the article and approved the submitted version.

## Funding

This work was supported by School of Psychology Massey University New Zealand.

## Conflict of Interest

The authors declare that the research was conducted in the absence of any commercial or financial relationships that could be construed as a potential conflict of interest.

## Publisher’s Note

All claims expressed in this article are solely those of the authors and do not necessarily represent those of their affiliated organizations, or those of the publisher, the editors and the reviewers. Any product that may be evaluated in this article, or claim that may be made by its manufacturer, is not guaranteed or endorsed by the publisher.

## Footnotes

^1^In NZ, the campaign Living wage rate is adjusted each year in response to cost of living, by the New Zealand Family Centre Social Policy Unit (King and Waldegrave, 2012, 2014).

^2^We are grateful to an informal ILO reviewer for this helpful section, which is included almost verbatim.

^3^We attribute this suggestion with appreciation to one of our expert peer reviewers, from whom it has been borrowed almost verbatim.

^4^We are grateful to one of our expert peer reviewers for this guiding future research suggestion.
